# Integrative oxidative stress–disulfidptosis analysis identifies *NPR3* and *ZNF366* as cell-type-associated candidate markers in clear cell renal cell carcinoma

**DOI:** 10.3389/fmolb.2026.1912537

**Published:** 2026-09-23

**Authors:** Ping Wang, Zhixuan Cai, Gaofeng Wu, Guangfeng Zhu

**Affiliations:** Department of Urology, Xi’an International Medical Center Hospital, Xi’an, Shaanxi, China

**Keywords:** disulfidptosis, oxidative stress, renal cell carcinoma, single-cell RNA-seq, SLC7A11, tumor immune microenvironment

## Abstract

Disulfidptosis is a regulated cell-death program that occurs in SLC7A11-high cells under glucose limitation, but its relationship with oxidative stress in clear cell renal cell carcinoma (ccRCC) remains unclear. We integrated 313 oxidative stress-related genes with 24 disulfidptosis-related genes in the original TCGA-KIRC analysis. Pearson screening (|r| ≥ 0.8 and nominal two-sided P < 0.05) identified 21 disulfidptosis-oxidative stress (DOS) genes, from which three reported DOS subtypes, 63 subtype-associated genes and a four-gene score comprising *ZNF366*, *EMCN*, *GRIK3* and *NPR3* were derived. In an updated GDC-derived TCGA-KIRC dataset, both k = 3 classifications showed method-dependent stability, with weaker stability for the available 58-of-63-gene solution. Outcome-blind centroid transfer assigned all 101 E-MTAB-1980 cases and reproduced several molecular and immune/stromal differences in GSE73731, although the hard clusters have not been independently validated for prognosis. In the atezolizumab-containing arms of IMmotion150, the within-cohort re-standardized four-gene score was lower in patients with clinical benefit and was associated with progression-free survival; the DOS21 module score was not associated with clinical benefit. The fixed four-gene score had modest internal discrimination (bootstrap optimism-corrected C-index, 0.697), and its transfer was sensitive to platform scaling and stage adjustment. Under the single 6-h glucose-deprivation condition, *NPR3* and *ZNF366* increased only in SLC7A11-high UMRC6 cells. Single-cell analyses in one VHL-related case and an independent 19-patient ccRCC cohort supported endothelial enrichment of *ZNF366* and showed that *NPR3* was not restricted to tumor cells. These findings identify *NPR3* and *ZNF366* as compartment-associated candidate markers but do not establish a functional role in disulfidptosis.

## Introduction

Disulfidptosis is a recently described form of regulated cell death whose execution depends on high expression of the cystine transporter SLC7A11 together with the cellular glucose state. When glucose is scarce, SLC7A11-high cells continue to import large amounts of cystine but cannot reduce it because NADPH is limiting; disulfide bonds then accumulate aberrantly, the actin cytoskeleton collapses, and the cell dies. This mode is distinct from apoptosis and ferroptosis and creates a potential metabolic vulnerability in SLC7A11-high, metabolically active tumors (Galluzzi et al., 2018; Tang et al., 2019; Dixon et al., 2012; Stockwell et al., 2017; Liu et al., 2023; Koppula et al., 2018).

How reactive oxygen species (ROS) relate to disulfidptosis is still unclear. ROS do not trigger disulfidptosis directly, but under metabolic stress they may aggravate redox imbalance and reshape the tumor microenvironment, thereby modifying susceptibility to SLC7A11-dependent disulfidptosis. Systematic studies of this oxidative stress–disulfidptosis interface remain scarce, and the molecules involved are poorly defined (Hayes et al., 2020; Sies et al., 2017; Sies and Jones, 2020; Schieber and Chandel, 2014).

Clear cell renal cell carcinoma (ccRCC), the histologic subtype represented by TCGA-KIRC, is characterized by metabolic reprogramming, including a dysregulated von Hippel–Lindau (VHL)–hypoxia-inducible factor (HIF) axis, altered lipid and glucose metabolism, neovascularization and oxidative stress (Vander Heiden et al., 2009; Pavlova and Thompson, 2016; Cancer Genome Atlas Research Network, 2013; Ricketts et al., 2018; Clark et al., 2019; Jonasch et al., 2021; Linehan et al., 2019; Wettersten et al., 2015; Kaelin, 2008; Chen et al., 2016). Its immune microenvironment can be active but dysfunctional (Senbabaoglu et al., 2016; Braun et al., 2020). These features provide a biological setting in which to examine the relationship between oxidative stress and disulfidptosis. Because the main bulk datasets are ccRCC cohorts, we use ccRCC for cohort-specific conclusions and reserve renal cell carcinoma (RCC) for broader biological or cell-line contexts.

In RCC, expression patterns may capture disulfidptosis-related variation that is not represented by recurrent genomic alterations. We therefore integrated oxidative stress-related and disulfidptosis-related genes and resolved the resulting candidates by cell type. This approach was intended to identify markers that would not be evident from mutation or single-gene analyses alone (Krishna et al., 2021; Bi et al., 2021; Young et al., 2018; Yu et al., 2023).

We integrated 313 oxidative stress-related genes with 24 disulfidptosis-related genes to construct a disulfidptosis–oxidative stress (DOS) framework in ccRCC. The framework was used for molecular subtyping and prognostic modeling, followed by sensitivity and transfer analyses of the reported classifications and risk score, treatment-context outcome analysis in IMmotion150, experiments in RCC cell lines, and cell-type localization in two single-cell cohorts. The study was designed to identify associations and candidate markers, not to establish causal regulation of disulfidptosis.

## Materials and methods

### Data sources and gene sets

Transcriptomic expression profiles, somatic mutations, copy-number variation and clinical follow-up for the original discovery analysis were obtained from the Cancer Genome Atlas Kidney Renal Clear Cell Carcinoma project (TCGA-KIRC) (Cancer Genome Atlas Research Network, 2013; Ricketts et al., 2018; Colaprico et al., 2016). Because the processed discovery matrix and analysis code were unavailable, subsequent analyses were treated as sensitivity or transfer analyses rather than an exact reconstruction. GDC-derived TCGA-KIRC STAR-TPM and survival matrices were retrieved from the UCSC Xena GDC hub on 17 July 2026 (Goldman et al., 2020; UCSC Xena, 2026). The expression matrix contains log2 (TPM+1)-transformed STAR-TPM values. It included 533 primary tumors, of which 529 had complete, valid overall-survival data. External analyses used E-MTAB-1980 (101 ccRCC cases, 23 deaths) (Sato et al., 2013; EMBL-EBI BioStudies, 2013), GSE73731 (265 ccRCC tumors) (Wei et al., 2017; NCBI Gene Expression Omnibus, 2017) and GSE207493 (19 ccRCC patients) (Yu et al., 2023; Lu et al., 2025; NCBI Gene Expression Omnibus, 2023). The IMmotion150 biomarker subset comprised 263 treatment-naive metastatic RCC tumors with baseline RNA-seq and outcome annotations, including 174 patients in atezolizumab-containing arms and 89 in the sunitinib arm (McDermott et al., 2018). A subsequent genomic study of the GSE207493 project described the 19 comparator samples as ccRCC without VHL germline mutation (Lu et al., 2025). We therefore refer to this cohort as a non-VHL-germline comparator; this designation does not prove that every case was sporadic or exclude other hereditary predisposition. Oxidative stress-related genes (OSRGs, n = 313) were compiled from published studies and oxidative stress-related Gene Ontology terms in MSigDB (Liberzon et al., 2011; Liberzon et al., 2015), principally GOBP_RESPONSE_TO_OXIDATIVE_STRESS (GO:0006979; systematic name M3223). Disulfidptosis-related genes (DRGs, n = 24) were collected from the original disulfidptosis study and subsequent work (Liu et al., 2023); the lists are provided in Supplementary Table S1. The discovery single-cell dataset GSE254444 comprised five ccRCC tumor lesions and one normal epithelial sample from one VHL-related renal cancer case (Barrett et al., 2013; Rowe et al., 2026; NCBI Gene Expression Omnibus, 2026).

### Identification of DOS genes

Pearson correlations were calculated between each OSRG and DRG in the original TCGA-KIRC analysis. Genes correlated with at least one member of the other set at |r| ≥ 0.8 and nominal two-sided P < 0.05 were retained, yielding 21 disulfidptosis–oxidative stress-related (DOS) genes. Multiplicity could not be reassessed for this original screening step because the processed matrix was unavailable; the screen is therefore considered exploratory. Somatic mutation and copy-number profiles were visualized with maftools and associated tools (Mayakonda et al., 2018). Figure 1 distinguishes the original discovery workflow from the additional sensitivity, transfer and patient-level localization analyses.

**FIGURE 1:**
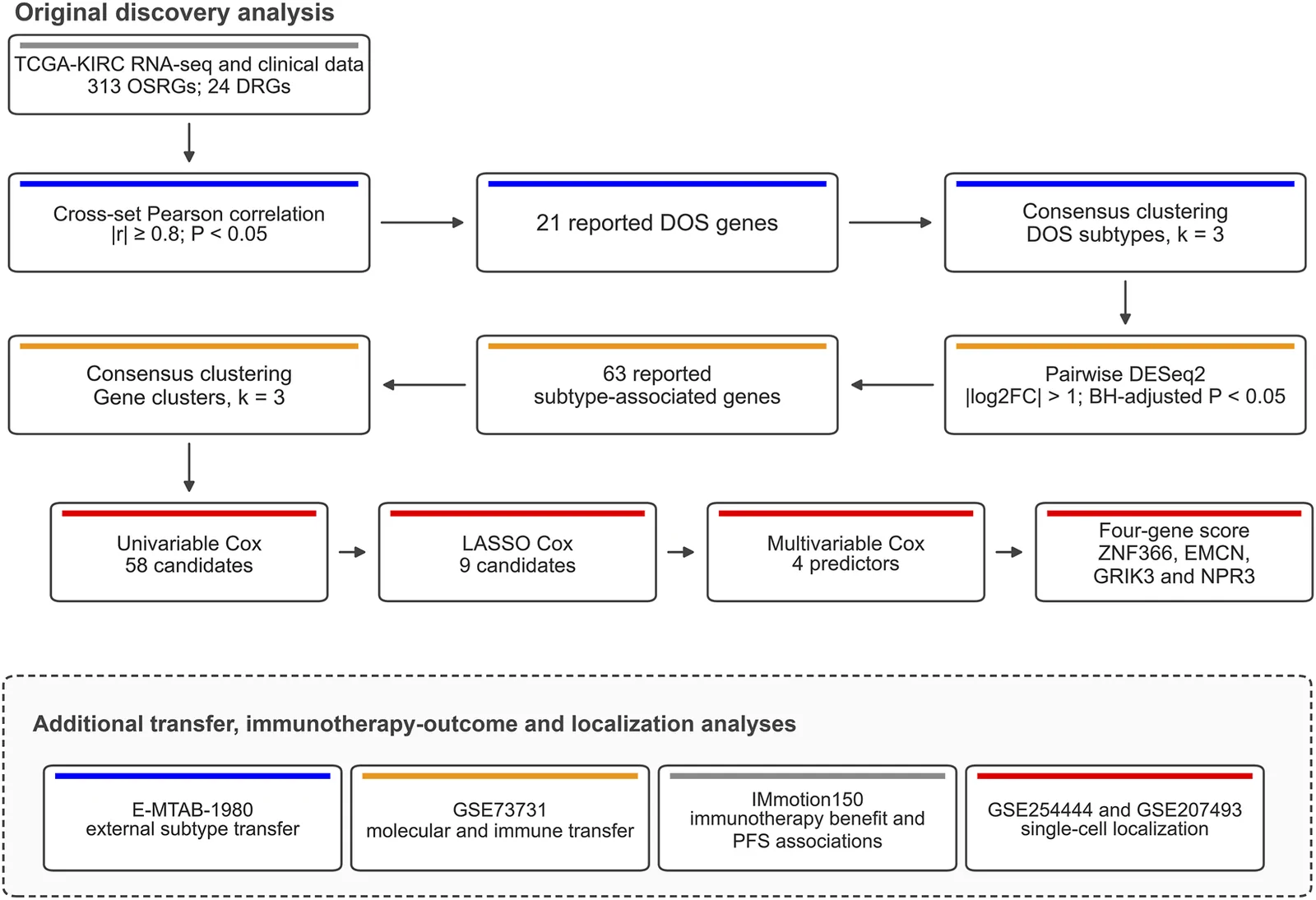
Study workflow. The main sequence summarizes the original TCGA-KIRC discovery analysis, from the curated OSRG and DRG sets through DOS screening, consensus clustering, subtype-associated differential expression and construction of the four-gene score. The lower dashed section summarizes the additional analyses: external subtype transfer in E-MTAB-1980, molecular and immune-pattern transfer in GSE73731, immunotherapy-context clinical-benefit and progression-free-survival associations in IMmotion150, and single-cell localization in GSE254444 and GSE207493. These analyses do not reconstruct the unavailable processed discovery dataset. DOS, disulfidptosis-oxidative stress; DRG, disulfidptosis-related gene; OSRG, oxidative stress-related gene.

### Molecular subtyping

Samples were grouped using the expression of the 21 DOS genes and unsupervised consensus clustering implemented in ConsensusClusterPlus (Wilkerson and Hayes, 2010). The reported k = 3 solution was selected from the consensus matrix and the cumulative distribution function (CDF) delta-area curve; subtype separation was examined by principal component analysis (PCA), and overall-survival differences were assessed using Kaplan–Meier curves and log-rank tests. The analogous gene-cluster analysis used the 63 genes shared across the subtype differential-expression comparisons.

### Cluster-stability and external molecular-phenotype transfer analyses

The original DOS and 63-gene classifications were evaluated in an updated GDC-derived TCGA-KIRC dataset without attempting to reproduce the unavailable discovery matrix. Stability was assessed across candidate values of k, by k = 3 resampling with 80% or 100% gene retention, alternative clustering algorithms, pairwise adjusted Rand indices (ARIs) and leave-one-gene-out analyses. All 21 DOS genes and 58 of the 63 subtype-associated genes were available. For external transfer, TCGA-derived, gene-wise standardized k = 3 centroids were fixed before E-MTAB-1980 and GSE73731 were evaluated, and each sample was assigned to the centroid with the highest Pearson correlation. This procedure is a surrogate transfer analysis, not an application of the unavailable original classifier. In E-MTAB-1980, overall survival across transferred groups was explored using global and pathological-T-stage-stratified log-rank tests. Because only 23 deaths were available and the findings were not robust to stage stratification, this analysis was treated as exploratory. In GSE73731, eleven prespecified immune and stromal scores were calculated as the mean of gene-wise z-standardized expression values and were not interpreted as estimated cell fractions. Scores and clinicopathological variables were compared with Kruskal-Wallis tests, with Benjamini-Hochberg correction applied separately to the immune/stromal and clinicopathological families. GSE73731 lacked usable overall- and progression-free-survival outcomes. These external analyses therefore evaluate subtype and immune-pattern transfer rather than prognostic validation (Figure 6; Supplementary Table S2).

### Differential expression and functional enrichment

Differentially expressed genes among DOS subtypes were identified with DESeq2 (Love et al., 2014) using |log2 fold change| > 1 and Benjamini–Hochberg (BH)-adjusted P (padj) < 0.05; the intersection of pairwise comparisons defined the reported 63-gene set. Gene Ontology (GO) and Kyoto Encyclopedia of Genes and Genomes (KEGG) enrichment analyses were performed with clusterProfiler (Wu et al., 2021), with multiple testing controlled by BH adjustment. Pathway activity was assessed with gene set variation analysis (GSVA) using Hallmark gene sets (Liberzon et al., 2015; Haenzelmann et al., 2013). In bulk TCGA-KIRC analyses, the disulfidptosis and oxidative-stress signature scores were calculated by GSVA from the DRG and OSRG sets, respectively.

### Prognostic risk model

Univariable Cox regression, least absolute shrinkage and selection operator (LASSO) Cox regression using glmnet (Friedman et al., 2010; Simon et al., 2011), and multivariable Cox regression were applied sequentially to the differential genes, yielding *ZNF366*, *GRIK3*, *EMCN* and *NPR3*. The reported risk score was defined as −0.3638×*ZNF366* −0.2996×*EMCN* +0.4730×*GRIK3* −0.1654×*NPR3*. Samples were randomly divided 1:1 into training and test sets. The original analyses evaluated Kaplan–Meier separation, time-dependent receiver operating characteristic curves, and univariable and multivariable Cox models including the risk score, sex, age, grade, and T, M and N stage. A coefficient in this multivariable score denotes a conditional association given the other predictors and is not interpreted as the gene’s unadjusted biological effect.

### Risk-model robustness and external overall-survival transfer

The four predictors and original coefficients were held fixed in analyses using the updated GDC-derived TCGA-KIRC dataset; upstream feature selection was not repeated. In the 529 complete primary-tumor cases, pairwise Pearson correlations and variance inflation factors (VIFs) were calculated. A VIF of 5 was prespecified only as a heuristic reference, not as a significance threshold. Coefficient stability was examined with 1,000 case-bootstrap resamples. Discrimination was assessed using the apparent C-index, bootstrap optimism correction and 20 repeats of five-fold cross-validation. External transfer was tested in E-MTAB-1980 using continuous scores to avoid imposing an unsupported cutoff. Cox models reported HRs per 1-SD higher score for the original array-scale formula and for a gene-wise standardized sensitivity score, before and after adjustment for pathological T stage. The standardized score tests sensitivity to platform scaling and is not proposed as a replacement clinical model. BH correction was applied across the four prespecified score models (Supplementary Figure S8).

### IMmotion150 outcome-association analysis

Baseline log2 (TPM+1) values in the 263-sample IMmotion150 biomarker subset were standardized gene-wise within the cohort. The four-gene score was then calculated as −0.3638 × *ZNF366* −0.2996 × *EMCN* + 0.4730 × *GRIK3* −0.1654 × *NPR3*. Because this procedure changes the original expression scale, the result was treated as a within-cohort re-standardized score rather than a locked external validation. The DOS21 module score was the mean standardized expression of the 21 DOS genes. Outcome analyses were restricted to the 174 patients in atezolizumab-containing arms; 165 had clinical-benefit annotations (113 with benefit and 52 without benefit), and all 174 had progression-free-survival data. Score distributions were compared using two-sided Wilcoxon rank-sum tests. BH correction was applied across four prespecified clinical-benefit comparisons (four-gene score, DOS21 score, *NPR3* and *ZNF366*). Progression-free survival was assessed with a continuous-score univariable Cox model and, descriptively, with a within-cohort median split, Kaplan–Meier curves and a log-rank test. No treatment-by-score interaction was tested; these analyses assess outcome associations within the ICI-treated subset and not treatment-predictive utility (Figures 6E–G).

### Immune microenvironment analysis

Immune-cell infiltration was estimated by single-sample gene set enrichment analysis (ssGSEA) (Subramanian et al., 2005; Barbie et al., 2009). The original exploratory analyses compared ssGSEA scores across subtypes and risk groups and examined correlations with the risk score and candidate genes. Tumor Immune Dysfunction and Exclusion (TIDE) scores were used as an exploratory estimate of potential immune-checkpoint-blockade response. Predicted half-maximal inhibitory concentration (IC50) values for selected Genomics of Drug Sensitivity in Cancer agents were compared between risk groups. These computational estimates do not demonstrate clinical treatment response.

### Single-cell transcriptome analysis

GSE254444 single-cell data were processed using Seurat (Stuart et al., 2019; Hao et al., 2021), with Harmony used for batch correction across lesions (Korsunsky et al., 2019). Quality control retained cells with 500–10,000 detected genes and mitochondrial transcript content <20%, yielding 26,184 cells (median, 1,802 genes). Following normalization, dimensionality reduction and clustering, cell types were annotated using canonical markers. AddModuleScore was used to calculate transcriptional disulfidptosis and oxidative-stress module scores from the DRG and OSRG sets. Because all five lesions came from one VHL-related case, cell-level correlations in this dataset are considered exploratory and do not provide independent patient-level replication.

### Patient-level external single-cell analysis

GSE207493 was used as an external patient-level single-cell cohort. For each of 19 ccRCC patients, log-normalized *NPR3* and *ZNF366* expression and the disulfidptosis-associated and oxidative-stress-associated transcriptional scores were averaged within tumor, endothelial and immune compartments before testing. Overall compartment differences were assessed by two-sided Friedman tests, with BH correction across four endpoints and Kendall’s W as effect size. Pairwise compartment comparisons used two-sided exact paired Wilcoxon signed-rank tests with BH correction across 12 comparisons. The patient, not the cell, was the biological replicate (Supplementary Figure S7). This analysis evaluates compartment-associated transcriptional localization and is not a direct measurement of ROS, disulfidptosis activity or mechanism.

### Cell culture and experimental validation

Human RCC cell lines UMRC6 and 786-O were authenticated by short tandem repeat profiling and used to contrast the observed SLC7A11-high UMRC6 and SLC7A11-low 786-O backgrounds (Figure 8A). This contrast is consistent with prior work showing greater and more rapid glucose-starvation sensitivity in SLC7A11-high UMRC6 cells than in parental 786-O cells (Liu et al., 2023). The retained laboratory record did not contain sufficient detail to reconstruct the basal culture medium and serum concentration. Cells were exposed to glucose-free Dulbecco’s modified Eagle medium (Gibco) for 6 h. A previous UMRC6 study detected non-reducing cytoskeletal disulfide migration after 6 h of glucose starvation (Wang et al., 2024), supporting this duration as an acute assay window. However, 6 h was the only condition retained for the present study and was not selected by a within-study time-course or dose-response experiment. *NPR3*, *ZNF366*, *EMCN* and *GRIK3* expression was measured by quantitative real-time polymerase chain reaction (qRT-PCR) in at least three biological replicates per group, with *GAPDH* as the reference and relative expression calculated using the 2^−ΔΔCt^ method; primers are listed in Table 1. SLC7A11 protein was detected by Western blotting with tubulin as the loading control. Drebrin (Proteintech) was resolved under reducing (R) and non-reducing (NR) conditions. Its high-molecular-weight disulfide-crosslinked form under NR conditions was used as a disulfidptosis-related cytoskeletal readout. No dose-response or time-course experiment was available.

**TABLE 1:** Primer sequences used for qRT-PCR validation of *GRIK3*, *ZNF366*, *NPR3* and *EMCN*.

Gene	Forward primer (5′-3′)	Reverse primer (5′-3′)
*GRIK3*	AGAAGTTGCTCCCCTCCAC	CCCCACACCCAGTACCC
*ZNF366*	GGGTCTAGGAAACGGAAGA	TGCAGGGTGGTTATAGGG
*NPR3*	ATGATGGTGTTCAGAGGGA	ATTCTTCTTGCTGAGTATGGC
*EMCN*	CACTACACCTTTTCCATTTGC	AGCTGCCTCTAAAACACCTG

All primer sequences are shown in the 5′-3′ direction.

### Statistical analysis

Statistical analyses were performed in R, and all tests were two-sided. Original two-group comparisons were reported as t tests or Wilcoxon rank-sum tests, but distributional checks and the exact test-selection decisions could not be reverified because the underlying values and source code were unavailable. Survival differences were assessed with Kaplan–Meier curves and log-rank tests; Cox models are reported with hazard ratios (HRs) and 95% confidence intervals (CIs). Pearson correlation was used for DOS screening and for the four-gene correlation matrix in the updated GDC-derived dataset. Original correlation, immune-infiltration and drug-sensitivity panels are treated as exploratory because their correlation choices and multiplicity procedures could not be fully recovered. For the additional analyses, multiplicity was controlled by BH adjustment within the prespecified families described above. Exact P values are reported when available; P < 0.05 was used as a descriptive threshold.

## Results

### Integrative analysis of oxidative stress and disulfidptosis genes identifies 21 DOS genes

Figure 1 summarizes the study design, separating the original TCGA-KIRC discovery sequence from the external transfer, immunotherapy-outcome and patient-level localization analyses. The original analysis integrated 313 OSRGs with 24 DRGs and retained 21 genes meeting the prespecified cross-set Pearson-correlation criteria. These genes formed the basis of three reported molecular subtypes, 63 subtype-associated genes and a four-gene score. The displayed additional branch comprises E-MTAB-1980, GSE73731, IMmotion150, GSE254444 and GSE207493; updated GDC-derived TCGA-KIRC data were used separately for sensitivity and fixed-model diagnostics. In the original data, the 21 DOS genes showed correlated expression patterns (Figures 2A–C), variable univariable survival associations (Figure 2D; Supplementary Figure S1), infrequent somatic mutation (23/379, 6.07%; Figure 2E) and limited copy-number variation (Figure 2F). Together, these observations motivated a multigene expression-based analysis.

**FIGURE 2:**
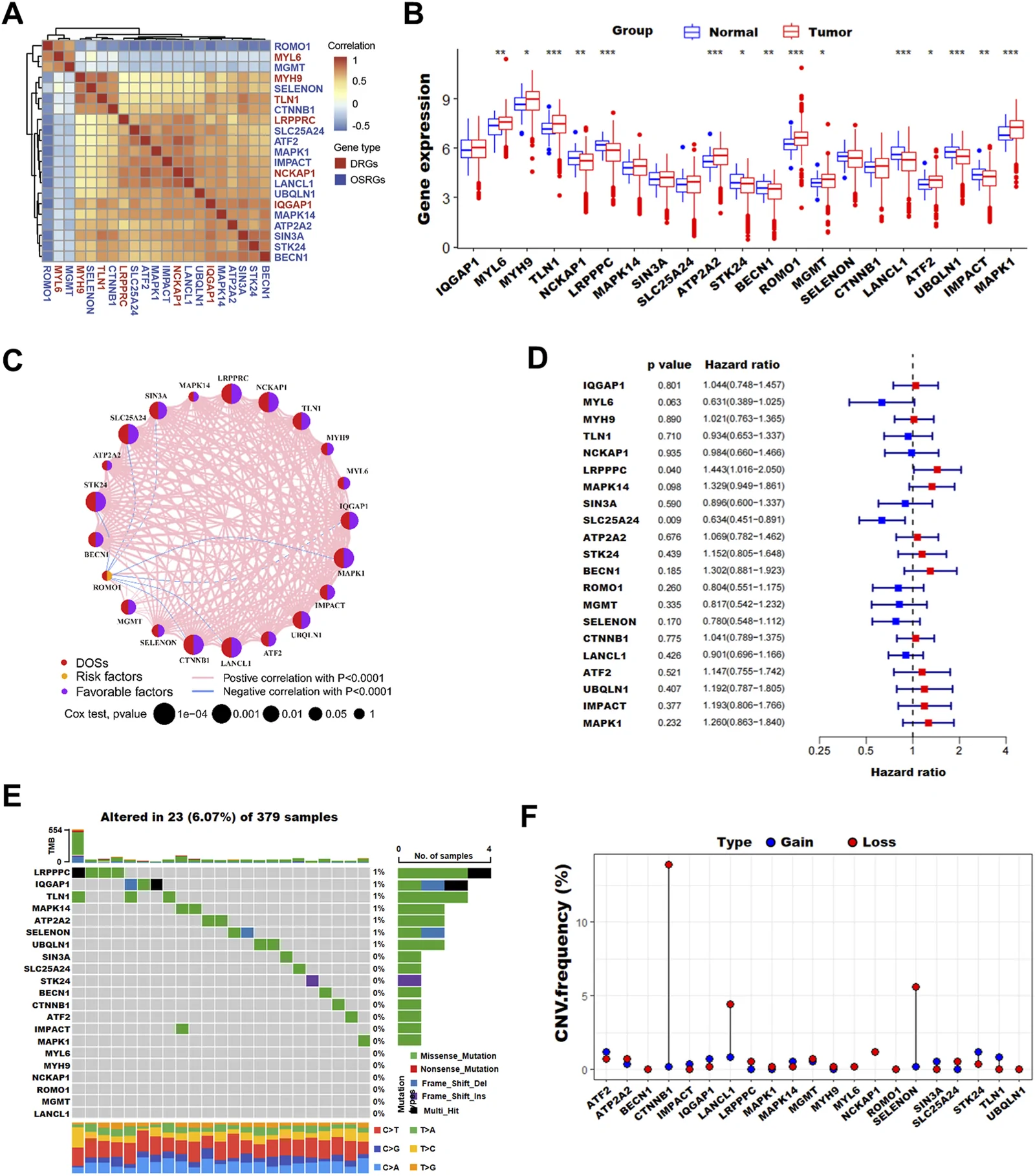
Screening and characterization of disulfidptosis–oxidative stress-related genes in clear cell renal cell carcinoma. **(A)** Correlation matrix of selected OSRGs and DRGs. **(B)** Expression distributions in normal and tumor tissues. **(C)** Correlation and prognostic-association network. **(D)** Univariable Cox regression for overall survival; points show HRs and horizontal lines show 95% CIs. **(E)** Somatic-mutation landscape. **(F)** Copy-number gain and loss frequencies. CI, confidence interval; DOS, disulfidptosis–oxidative stress; DRG, disulfidptosis-related gene; HR, hazard ratio; OSRG, oxidative stress-related gene.

### DOS-based molecular subtyping reveals differences in disulfidptosis-related features, prognosis and the immune microenvironment

Consensus clustering of the 21 DOS genes produced the reported three-group solution (Figures 3A,B), and PCA showed separation in expression space (Figure 3C). The subtypes differed in DOS-gene expression (Figure 3D) and overall survival (log-rank P < 0.001), with subtype B showing the most favorable survival (Figure 3E). Immune-cell scores also differed among groups (Figure 3F). The simultaneous presence of inflammatory and regulatory populations in subtype B indicates a mixed immune landscape rather than uniformly enhanced antitumor immunity. Stability and transferability of this reported partition were evaluated separately below.

**FIGURE 3:**
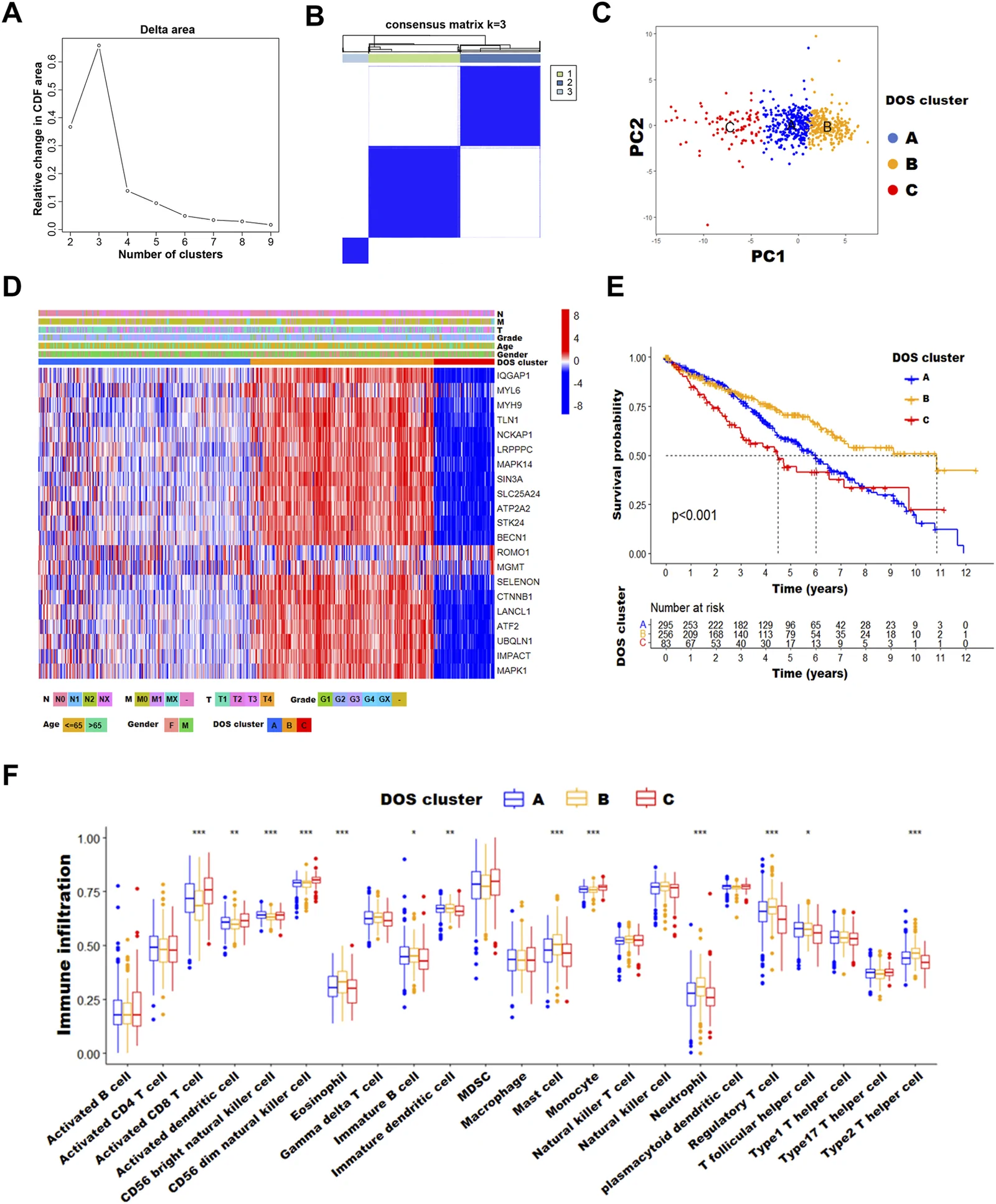
DOS-based molecular subtypes and their clinical and immune features in the original TCGA-KIRC analysis. **(A)** CDF delta-area curve used to evaluate candidate cluster numbers. **(B)** Consensus matrix for the reported k = 3 DOS solution. **(C)** PCA of the three DOS subtypes. **(D)** DOS-gene expression and clinicopathological annotations across subtypes. **(E)** Kaplan-Meier overall-survival curves; groups were compared with a two-sided log-rank test. **(F)** ssGSEA immune-cell scores across DOS subtypes. The underlying values and source code for the archived immune panel were unavailable, so its original test selection and multiplicity procedure could not be reverified; displayed P values are interpreted as exploratory. CDF, cumulative distribution function; DOS, disulfidptosis–oxidative stress; PCA, principal component analysis; ssGSEA, single-sample gene set enrichment analysis.

### Analyses in the updated GDC dataset show method-dependent cluster stability and mixed transfer to external cohorts

In the updated GDC-derived dataset, the DOS k = 3 solution had a PAC of 0.568 and mean silhouette of 0.304, whereas the 58-of-63-gene solution had a PAC of 0.764 and mean silhouette of 0.115 (Figure 6B). Alternative algorithms and leave-one-gene-out analyses showed moderate and, for several Gene58 comparisons, low agreement (Supplementary Table S2). Overall survival differed among the same-cohort DOS groups (log-rank P = 0.00262, BH q = 0.00262) and Gene58 groups (P = 1.14 × 10^−6^, q = 2.29 × 10^−6^), but these sensitivity results are not external validation. Outcome-blind centroid transfer assigned all 101 E-MTAB-1980 tumors, with group sizes of 33/25/43 for DOS21 and 20/44/37 for Gene58 (Figure 6A). Survival comparisons among these transferred groups were limited by 23 deaths and were not robust to pathological-T-stage stratification, so they were not interpreted as prognostic validation. GSE73731 retained 20 of 21 DOS genes and 56 of 63 subtype-associated genes; transferred group sizes were 93/80/92 for DOS21 and 62/103/100 for Gene58 (Figure 6A). After BH correction, 9 of 11 and 11 of 11 immune/stromal signatures differed across the respective classifications, while stage and grade did not (Figures 6C,D). The A-C labels are display labels for the transferred groups and do not imply one-to-one correspondence with the archived groups. Together, these findings support subtype assignment in two external cohorts and transfer of several immune/stromal patterns in GSE73731, but they do not establish external prognostic validation.

### Subtype differences converge on angiogenesis and extracellular matrix remodeling

Pairwise differential-expression analysis among DOS subtypes yielded 63 genes shared across comparisons (Figure 4A). These genes produced a reported three-group solution (Figures 4B,C) that separated by PCA (Figure 4D) and differed in overall survival (Figure 4E). Expression, clinicopathological and immune patterns are shown in Figure 4F and Supplementary Figure S2. GO and KEGG enrichment highlighted angiogenesis, extracellular-matrix organization, focal adhesion, and PI3K–Akt, Rap1 and Ras signaling (Figures 4G,H). These enrichment results support an association with endothelial and matrix-related processes but do not identify a causal pathway.

**FIGURE 4:**
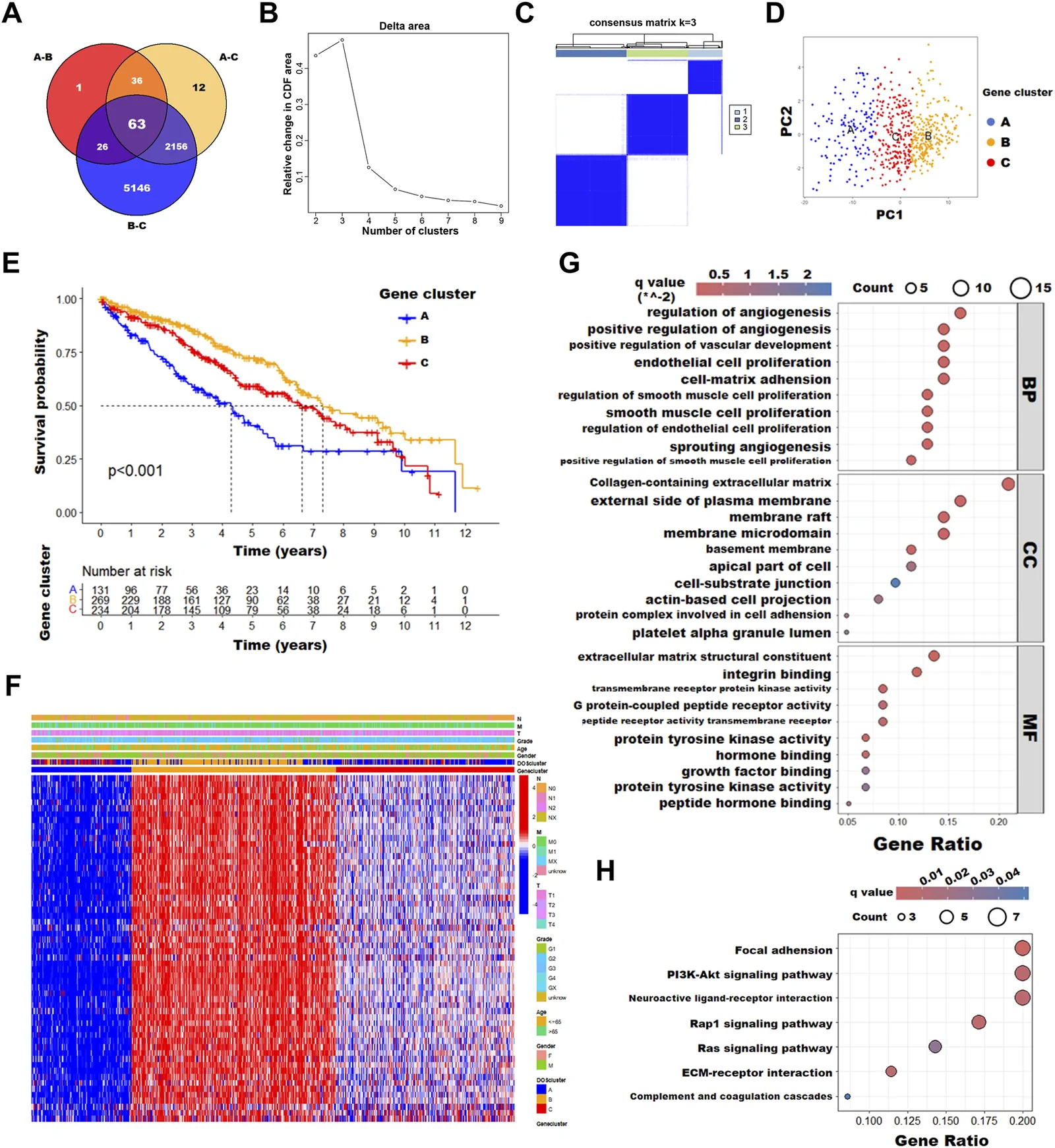
Identification and characterization of genes associated with the DOS subtypes in the original TCGA-KIRC analysis. **(A)** Intersection of genes meeting |log2 fold change| > 1 and DESeq2 BH-adjusted P < 0.05 in the pairwise subtype comparisons. **(B)** CDF delta-area curve for clustering based on the reported 63-gene set. **(C)** Consensus matrix for the reported k = 3 gene-cluster solution. **(D)** PCA of gene clusters. **(E)** Kaplan–Meier overall-survival curves; groups were compared with a two-sided log-rank test. **(F)** Expression of the 63 genes and clinicopathological annotations. **(G)** GO and **(H)** KEGG enrichment; enrichment P values were adjusted by the BH method. CDF, cumulative distribution function; DOS, disulfidptosis–oxidative stress; GO, Gene Ontology; KEGG, Kyoto Encyclopedia of Genes and Genomes; PCA, principal component analysis.

### Prognostic modeling narrows the differential genes to four candidate genes

Sequential univariable Cox, LASSO and multivariable Cox analyses reduced the candidate set to *ZNF366*, *GRIK3*, *EMCN* and *NPR3* (Figure 5A; Supplementary Figure S3). The expression patterns of the four model genes across risk and clinical strata are shown in Figure 5B. The original score separated high- and low-risk groups in the training set, test set and full cohort (all displayed log-rank P < 0.001; Figure 5C). Additional exploratory associations of the risk score with clinicopathological stage, immune features, predicted drug sensitivity and TIDE are shown in Supplementary Figures S4A–F. The score was associated with outcome after adjustment for the available clinicopathological variables (Supplementary Figures S4G,H). Expression of the broader DOS gene set differed between the high- and low-risk groups (Figure 5D), and individual-gene survival analyses for the four model genes are shown in Figure 5E. The expression patterns of *NPR3* and *ZNF366* across the reported DOS subtypes and gene clusters are shown in Supplementary Figure S5. Higher *GRIK3* expression was associated with better survival in the univariable analysis, whereas its coefficient was positive in the four-gene model. This sign reversal is a conditional association: the coefficient describes *GRIK3* after adjustment for *ZNF366*, *EMCN* and *NPR3* and does not imply that *GRIK3* is biologically harmful. Additional correlation, collinearity and bootstrap analyses examined the stability of this conditional estimate.

**FIGURE 5:**
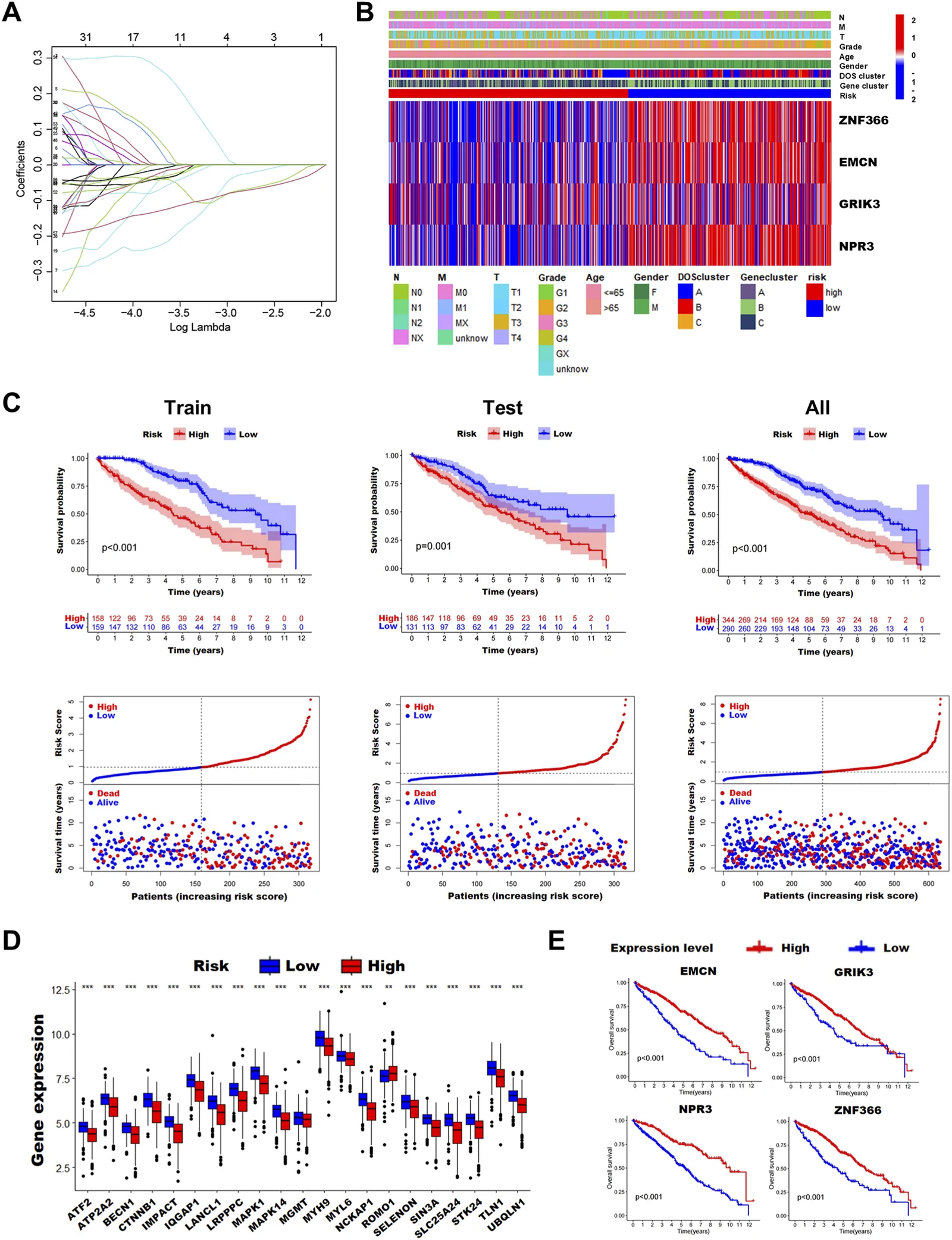
Construction and original evaluation of the four-gene DOS risk model. **(A)** LASSO Cox coefficient path. **(B)** Expression of *ZNF366*, *EMCN*, *GRIK3* and *NPR3* with clinical and molecular annotations. **(C)** Kaplan–Meier curves and risk distributions in the training, test and full cohorts; high- and low-risk groups were compared by two-sided log-rank tests. **(D)** DOS-gene expression by risk group. **(E)** Kaplan-Meier analyses for the four model genes. The original individual-level matrix, split seed and cutoff record were unavailable for rechecking; these panels are therefore reported as original analyses and are not used as external validation. DOS, disulfidptosis–oxidative stress; LASSO, least absolute shrinkage and selection operator.

### The fixed four-gene score shows modest discrimination and external transfer that depends on score scaling

In the updated GDC-derived complete-case cohort (n = 529), pairwise Pearson correlations among the four predictors ranged from r = 0.584 to 0.751, indicating moderate gene-gene collinearity; VIFs ranged from 1.984 to 3.049 (Supplementary Figures S8B,C). Across 1,000 fixed-model bootstrap resamples, median coefficients retained their original directions: −0.406 for *ZNF366* (95% percentile interval, −0.685 to −0.129), −0.393 for *EMCN* (−0.655 to −0.162), 0.535 for *GRIK3* (0.263–0.856) and −0.320 for *NPR3* (−0.508 to −0.131; Supplementary Figure S8D). This stability concerns conditional statistical associations and has no mechanistic interpretation. The apparent C-index was 0.703 and the optimism-corrected C-index was 0.697. Across 100 held-out folds from 20 repeats of five-fold cross-validation, the median C-index was 0.697 (2.5th–97.5th percentiles, 0.623–0.766; Supplementary Figure S8E). In E-MTAB-1980 (n = 101, 23 deaths), the original-scale score was associated with overall survival before adjustment (HR per 1-SD, 1.704; 95% CI, 1.084–2.678; BH q = 0.0417) but not after adjustment for pathological T stage (HR, 1.497; 95% CI, 0.979–2.288; q = 0.105). The gene-wise standardized sensitivity score remained associated in both models (unadjusted HR, 2.133; q = 1.29 × 10^−5^; adjusted HR, 1.775; q = 0.00178; Supplementary Figure S8F). The stronger standardized-score association indicates sensitivity to platform scaling, not superiority of a replacement model.

### Within-ICI-arm outcome associations in IMmotion150

Among patients in the atezolizumab-containing arms, the re-standardized four-gene score was lower in the clinical-benefit group than in the no-benefit group (n = 113 versus 52; two-sided Wilcoxon P = 0.00159, BH FDR = 0.00317), whereas the DOS21 module score did not differ (P = 0.791, FDR = 0.791). *NPR3* and *ZNF366* expression levels were higher in the clinical-benefit group (*NPR3*: P = 1.64 × 10^−5^, FDR = 6.56 × 10^−5^; *ZNF366*: P = 0.00789, FDR = 0.0105; Figure 6G). A higher continuous four-gene score was associated with shorter progression-free survival (HR per one-unit increase, 1.49; 95% CI, 1.12–1.98; Cox P = 0.00598). A descriptive within-cohort median split gave a log-rank P value of 0.0394 (Figure 6F). These findings provide treatment-context evidence for the continuous score and candidate genes but do not validate the DOS21 or Gene58 hard clusters, distinguish prognostic from treatment-specific association or establish an ICI-response classifier.

**FIGURE 6:**
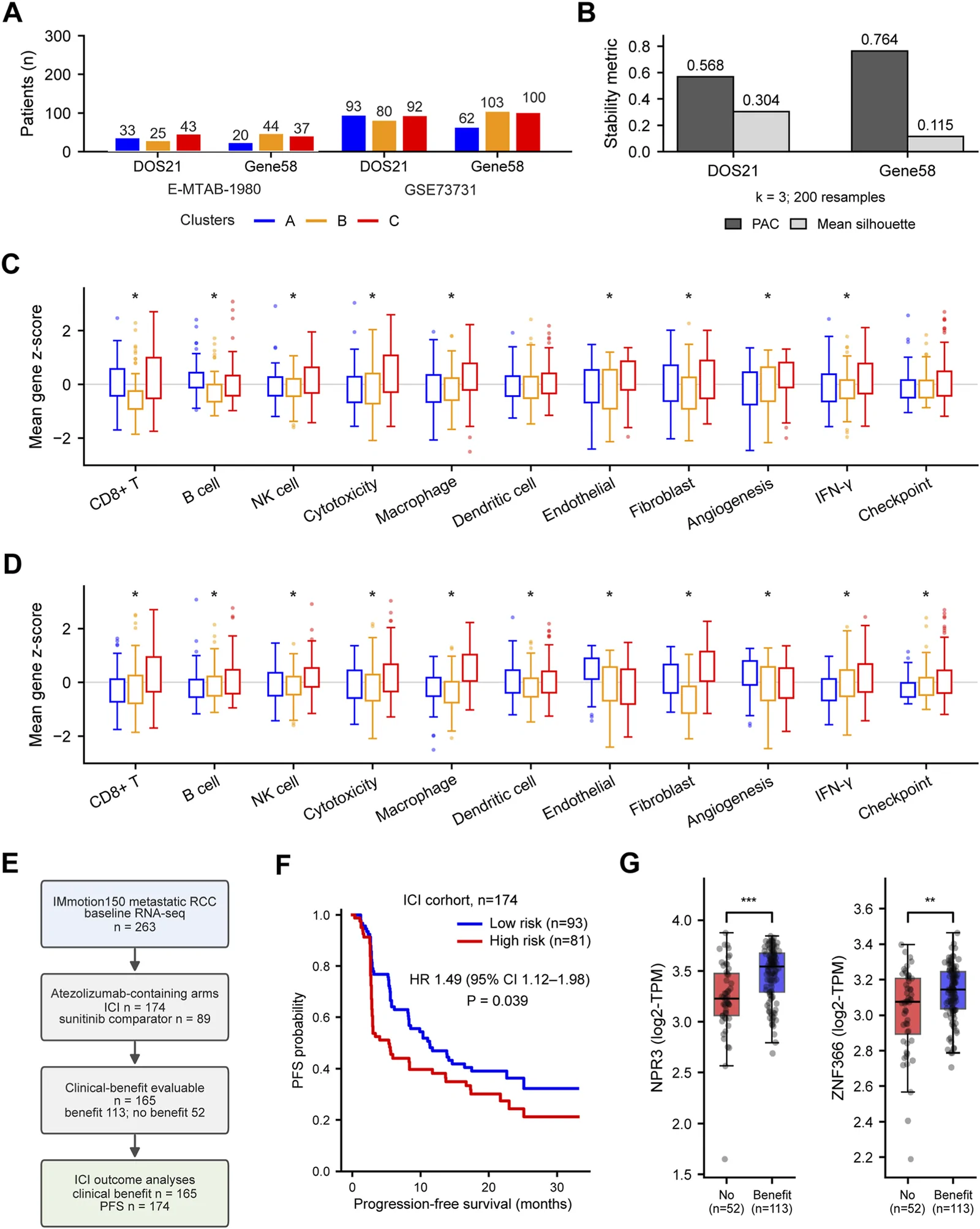
Cluster stability, external subtype and immune-pattern transfer, and IMmotion150 outcome associations. **(A)** Patient numbers assigned to display groups A, B and C after outcome-blind centroid transfer to E-MTAB-1980 and GSE73731. These display labels do not establish one-to-one correspondence with the archived A-C groups because the original processed matrix was unavailable. **(B)** PAC and mean silhouette values for the updated-GDC k = 3 DOS21 and Gene58 solutions from 200 consensus-clustering iterations with 80% sample and gene subsampling. These descriptive stability metrics are not replicate-level observations, so no inferential P value was assigned. **(C,D)** Per-patient immune and stromal signature scores in GSE73731 after outcome-blind centroid transfer of the DOS21 and Gene58 classifications, respectively. Each score is the mean of gene-wise standardized expression values and is not an estimated cell fraction. Boxes show medians and interquartile ranges; whiskers extend to 1.5 times the interquartile range. Asterisks indicate BH q < 0.05 for Kruskal-Wallis tests across three groups, corrected across 11 signatures within each classification. **(E)** IMmotion150 cohort accounting. **(F)** Progression-free survival after a within-cohort median split of the four-gene score in the atezolizumab-containing arms. The hazard ratio is from the continuous-score Cox model (HR 1.49, 95% CI 1.12–1.98; Cox P = 0.00598), whereas the displayed P = 0.039 is the median-split log-rank result. **(G)** *NPR3* and *ZNF366* expression by clinical benefit in the atezolizumab-containing arms. Boxes show medians and interquartile ranges; whiskers extend to 1.5 times the interquartile range, and points represent patients. Two-sided Wilcoxon rank-sum tests with BH correction gave P = 1.64 × 10^−5^ and FDR = 6.56 × 10^−5^ for *NPR3*, and P = 0.00789 and FDR = 0.0105 for *ZNF366*. The IMmotion150 analyses are retrospective within-treatment-arm associations; they do not validate the DOS21 or Gene58 hard clusters or establish treatment-predictive utility.

### Lower-risk tumors show higher computational immune-signature scores

The DOS-high subtype and gene cluster B mapped predominantly to the lower-risk group and better survival in the original analysis (Figures 7A,B). Tumor mutational burden differed modestly between risk groups (Figure 7C), and the lower-risk group had higher scores for several interferon and inflammatory Hallmark pathways (Figure 7D). GO and KEGG analyses also highlighted transport, metabolic and extracellular-matrix processes (Figures 7E,F). These results describe transcriptional immune and pathway features; they neither demonstrate immune activation *in vivo* nor predict clinical response to immunotherapy.

**FIGURE 7:**
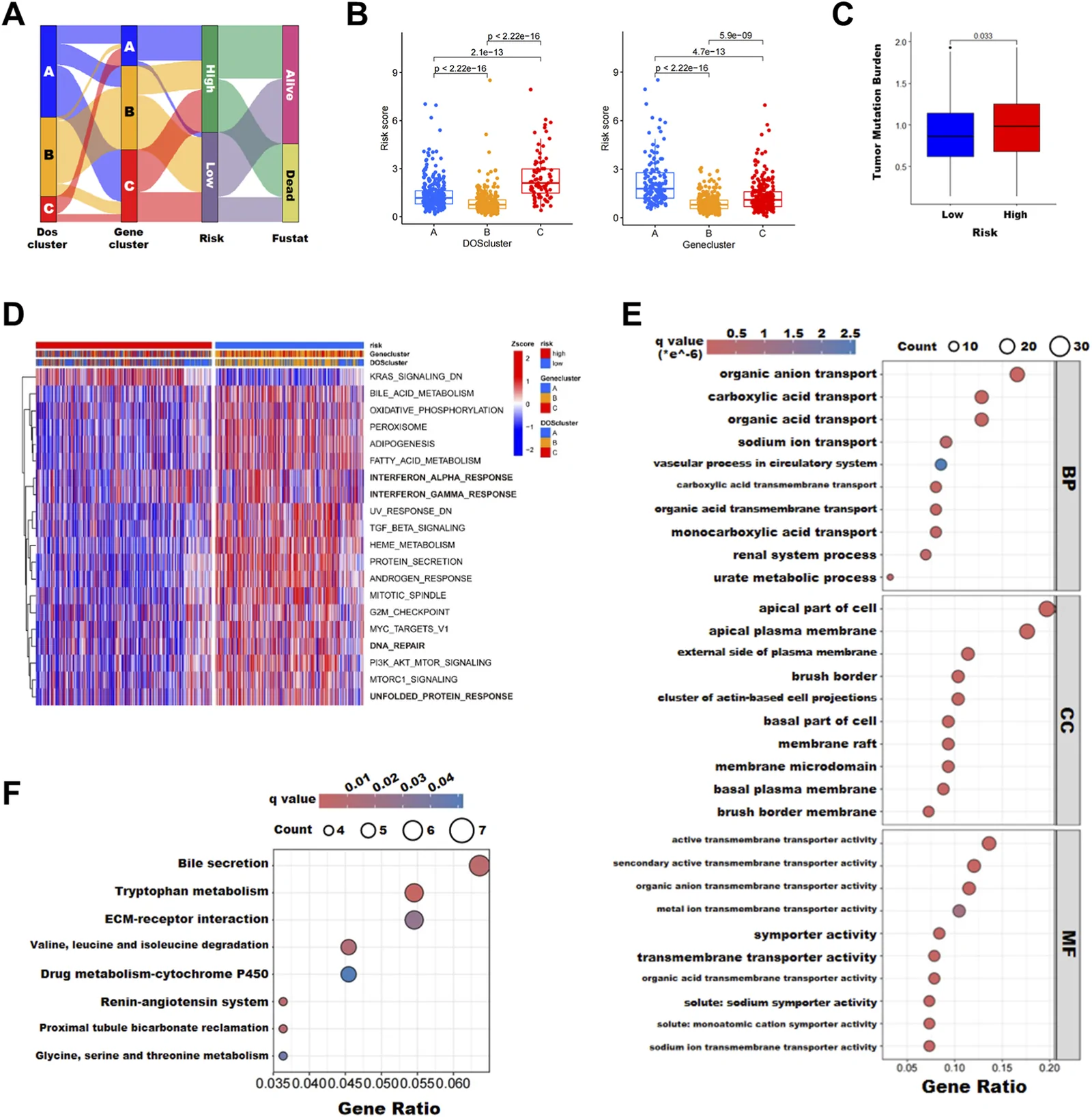
Associations among DOS subtypes, gene clusters, risk groups and functional features in the original TCGA-KIRC analysis. **(A)** Sankey diagram linking the classifications and survival status. **(B)** Risk-score distributions across DOS subtypes and gene clusters. **(C)** Tumor mutational burden by risk group. **(D)** GSVA heatmap of Hallmark pathway scores. **(E)** GO and **(F)** KEGG enrichment of genes differing between risk groups. The underlying complete test families were unavailable for rechecking; displayed group-comparison and enrichment P values are interpreted as exploratory. DOS, disulfidptosis–oxidative stress; GO, Gene Ontology; GSVA, gene set variation analysis; KEGG, Kyoto Encyclopedia of Genes and Genomes.

### Under the tested condition, glucose deprivation increases *NPR3* and *ZNF366* only in SLC7A11-high UMRC6 cells

UMRC6 showed higher SLC7A11 protein abundance than 786-O (Figure 8A) and a more evident Drebrin disulfide-crosslinking pattern during glucose deprivation in the original blot (Figure 8B). Under the single 6-h condition, *NPR3* and *ZNF366* increased in UMRC6 (P < 0.05), whereas *EMCN* and *GRIK3* did not; none of the four genes changed significantly in 786-O (Figures 8C,D). The underlying qRT-PCR values, exact P values and dispersion definition were unavailable for independent reanalysis. In bulk TCGA-KIRC, *NPR3* was not correlated with *SLC7A11* (r = 0.0099, P = 0.82), whereas *ZNF366* showed a weak positive correlation (r = 0.26, P = 5.8 × 10^−10^; Figure 8E). Both genes were positively correlated with the transcriptional disulfidptosis score (*NPR3*: r = 0.24, P = 1.3 × 10^−9^; *ZNF366*: r = 0.20, P = 1.1 × 10^−6^; Figure 8F). These data link *NPR3* and *ZNF366* to the tested stress condition but do not show that either gene induces, mediates or rescues disulfidptosis.

**FIGURE 8:**
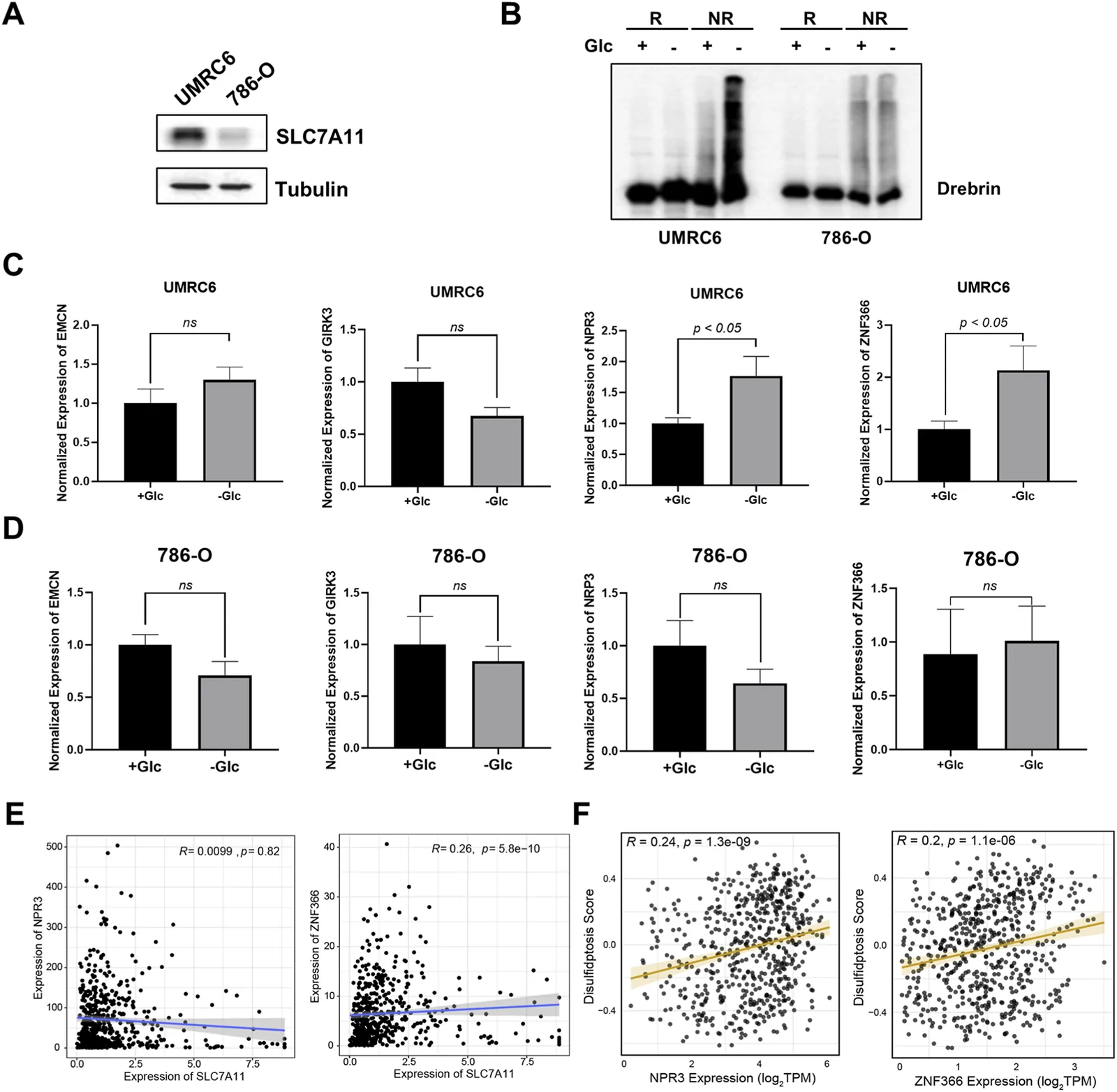
Experimental and transcriptomic assessment of *NPR3* and *ZNF366* in disulfidptosis-related contexts. **(A)** SLC7A11 protein abundance in UMRC6 and 786-O cells. **(B)** Drebrin under reducing (R) and non-reducing (NR) conditions with or without 6-h glucose deprivation. **(C,D)** qRT-PCR analysis of *EMCN*, *GRIK3*, *NPR3* and *ZNF366* after 6-h glucose deprivation in UMRC6 and 786-O, respectively. Bars and error bars reproduce the original summary display. The underlying qRT-PCR values were unavailable, so the dispersion definition, exact sample counts beyond the retained record of at least three biological replicates per group, and exact P values could not be independently recovered. **(E)** Pearson correlations of NPR3 and ZNF366 expression with *SLC7A11* in TCGA-KIRC. **(F)** Pearson correlations with the transcriptional disulfidptosis score. Glc, glucose; NR, non-reducing; qRT-PCR, quantitative real-time polymerase chain reaction; R, reducing. *P < 0.05; ns, not significant.

### Discovery single-cell analysis associates *NPR3* and *ZNF366* with tumor and endothelial compartments, respectively

The discovery single-cell analysis included 26,184 cells from five lesions derived from one VHL-related renal cancer case and identified tumor, endothelial, fibroblast, myeloid, T, B and natural killer cells (Figures 9A–C; Supplementary Figure S6). Tumor and other non-immune cells showed higher transcriptional disulfidptosis scores, and tumor cells showed the highest oxidative-stress module score (Figure 9D). Within tumor cells, the two module scores were positively correlated (r = 0.21, P < 2.2 × 10^−16^; Figure 9E). *NPR3* was most evident in tumor cells, whereas *ZNF366* was enriched in endothelial cells (Figure 9F); cell-level expression correlated with disulfidptosis scores in the corresponding compartments (both r = 0.16, P < 2.2 × 10^−16^; Figures 9G,H). Because cells were nested within a single individual, these P values reflect exploratory cell-level associations and not patient-level replication.

**FIGURE 9:**
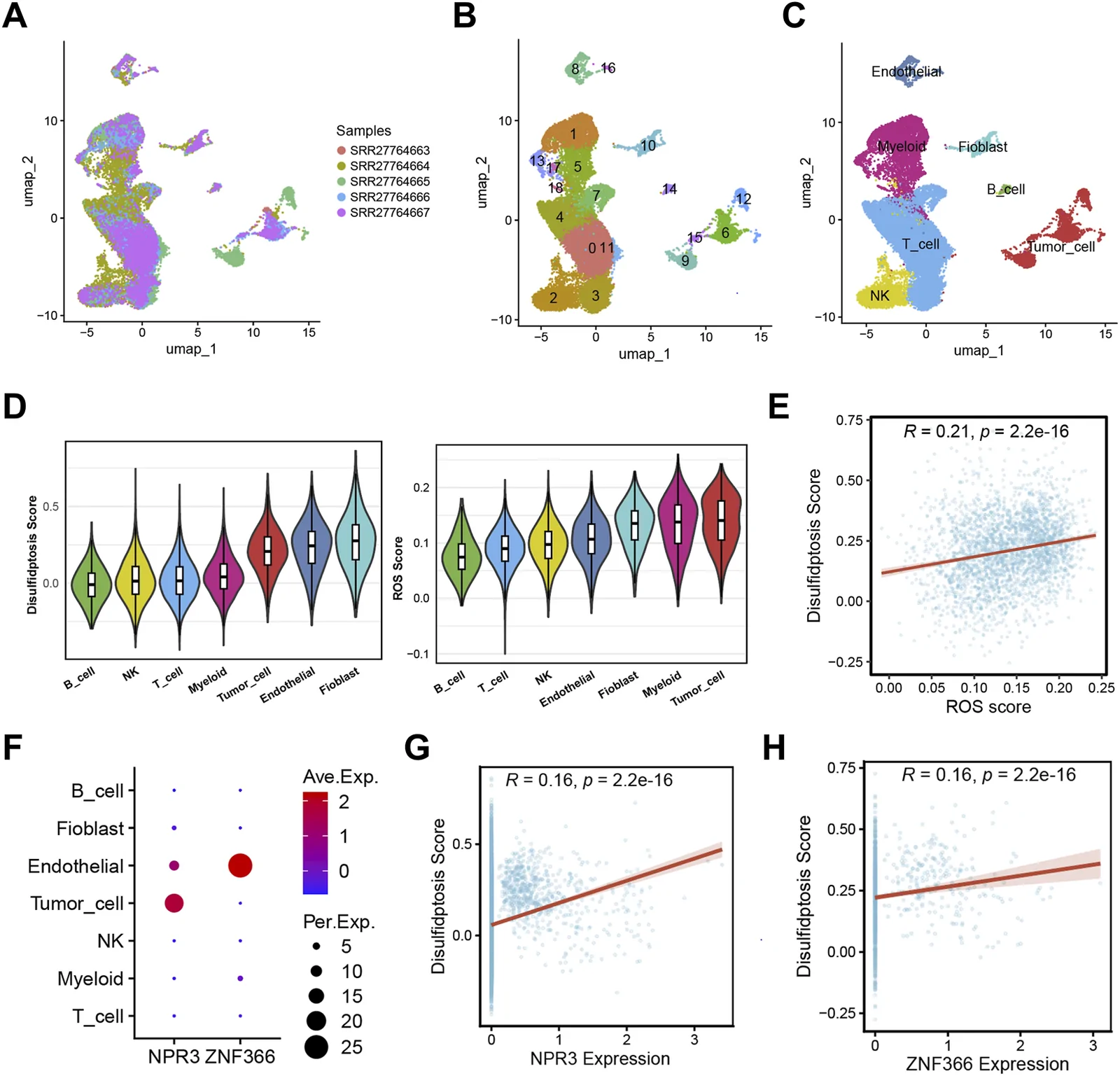
Discovery single-cell localization of *NPR3* and *ZNF366* and association with transcriptional disulfidptosis-related features. **(A)** UMAP by lesion. **(B)** UMAP by cluster. **(C)** Annotation of tumor, endothelial, fibroblast, myeloid, T, B and natural killer cells. **(D)** AddModuleScore-derived disulfidptosis and oxidative-stress scores by cell type. **(E)** Cell-level association between the two scores in tumor cells. **(F)** *NPR3* and *ZNF366* expression across cell types. **(G)** *NPR3* expression versus the disulfidptosis score in tumor cells. **(H)** *ZNF366* expression versus the disulfidptosis score in endothelial cells. The 26,184 cells were derived from five lesions from one VHL-related case. The original correlation implementation could not be independently recovered from the original code; these cell-level associations are exploratory and do not constitute patient-level replication. UMAP, uniform manifold approximation and projection.

### Patient-level external analysis supports endothelial *ZNF366* and broadens *NPR3* localization

Across 19 ccRCC patients in GSE207493, *ZNF366*, *NPR3* and the two transcriptional module scores differed among tumor, endothelial and immune compartments (Supplementary Figure S7). All Friedman BH-adjusted q values were ≤ 1.74 × 10^−6^, with Kendall’s W ranging from 0.698 to 0.950. *ZNF366* was higher in endothelial cells than in tumor or immune cells (both pairwise q = 6.54 × 10^−6^). *NPR3* was higher in tumor and endothelial cells than in immune cells (both q = 6.54 × 10^−6^), but did not differ between tumor and endothelial compartments (q = 0.953). The disulfidptosis-associated score was highest in endothelial cells, whereas the oxidative-stress-associated score was highest in tumor cells. Thus, the patient-level analysis supports endothelial enrichment of *ZNF366* and non-immune enrichment of *NPR3*, while showing that *NPR3* is not tumor-cell specific. These transcriptional scores are not direct measurements of disulfidptosis or ROS.

## Discussion

Disulfidptosis arises from disulfide stress in glucose-limited, SLC7A11-high cells and is mechanistically distinct from other regulated cell-death programs (Liu et al., 2023; Koppula et al., 2018). In the metabolically reprogrammed setting of ccRCC (Cancer Genome Atlas Research Network, 2013; Ricketts et al., 2018; Clark et al., 2019; Jonasch et al., 2021; Linehan et al., 2019; Wettersten et al., 2015; Kaelin, 2008; Chen et al., 2016), the oxidative stress–disulfidptosis transcriptional state was associated with molecular groupings, a four-gene prognostic signal and cell-type-specific expression patterns. *NPR3* was enriched outside immune cells, whereas *ZNF366* showed a reproducible endothelial pattern. These findings support their use as compartment-associated candidate markers; their functional roles in disulfidptosis remain to be determined.

The additional cluster analyses qualify the separation seen in Figures 3, 4. In the updated GDC-derived dataset, the DOS k = 3 solution had moderate stability, while the 58-of-63-gene solution had higher PAC, lower silhouette and weaker agreement across several alternative methods. Outcome-blind centroid transfer assigned the DOS21 and Gene58 groups in both E-MTAB-1980 and GSE73731. GSE73731 reproduced several molecular and immune/stromal differences but lacked usable survival outcomes, whereas the E-MTAB-1980 survival comparison was limited by 23 deaths and was not robust to pathological-T-stage stratification. The hard clusters are therefore best viewed as method-dependent exploratory molecular groupings rather than externally validated prognostic subtypes.

Analyses in the updated GDC-derived dataset clarify the *GRIK3* coefficient without assigning it a biological direction. Predictor correlations were moderate even though all VIFs were below the heuristic value of 5. The positive *GRIK3* coefficient was retained in all 1,000 fixed-model bootstrap resamples, which is compatible with a conditional or suppression-type association rather than a data-entry error. It does not rule out collinearity or imply a causal adverse effect. Both the optimism-corrected and cross-validated C-indices were approximately 0.697, indicating modest discrimination.

Continuous-score transfer to E-MTAB-1980 provided limited external support. The original-scale score was associated with overall survival before adjustment (HR per 1-SD, 1.70; 95% CI, 1.08–2.68; BH-adjusted P = 0.0417). The association was not significant after adjustment for pathological T stage (HR, 1.50; 95% CI, 0.98–2.29; adjusted P = 0.105). The gene-wise standardized score remained associated with survival in both models, although this cross-platform construction differs from the original formula. Its stronger association points to sensitivity to platform scaling and should not be interpreted as support for a replacement clinical model. The data therefore support transfer of a continuous four-gene signal, but not a validated cutoff or clinical risk classifier; prospective evaluation against established clinical models is required before clinical use.

IMmotion150 adds treatment-context evidence for the continuous four-gene signal. Patients with clinical benefit had lower re-standardized scores, and higher scores were associated with shorter progression-free survival in the atezolizumab-containing arms. The null DOS21 result is also informative: the broader module did not track clinical benefit in this dataset. Because the analysis used within-cohort standardization and did not test a treatment-by-score interaction against the sunitinib arm, it cannot separate prognostic from treatment-specific association or define a deployable cutoff.

The independent GSE207493 cohort clarifies the cell-type interpretation. A later genomic study of the same project described the 19 comparator samples as ccRCC without VHL germline mutation (Yu et al., 2023; Lu et al., 2025). This supports a non-VHL-germline comparison but cannot confirm that every case was sporadic or rule out other hereditary predisposition. Patient-level analyses confirmed marked endothelial enrichment of *ZNF366* and higher oxidative-stress scores in tumor than immune compartments. *NPR3 * was higher in both tumor and endothelial compartments than in immune cells, but tumor and endothelial levels did not differ (BH-adjusted P = 0.953). *NPR3* should therefore not be described as tumor-cell specific. The tumor cell-line response of *ZNF366* and its endothelial localization in patient samples concern different compartments; endothelial-specific function was not tested.

This study has several limitations. The processed discovery matrix and original code were unavailable, so the updated-data analyses do not reproduce feature selection, and the model diagnostics treat the four predictors as fixed. The hard clusters have not been independently validated for prognosis: GSE73731 lacked usable survival outcomes, and an exploratory E-MTAB-1980 analysis was limited by 23 deaths and was not robust to pathological T-stage stratification. The IMmotion150 analysis used a within-cohort re-standardized score in a single retrospective biomarker subset; without a treatment-by-score interaction, it cannot establish treatment-predictive utility. The cell-line experiment used two lines and one 6-h glucose-deprivation condition; no dose-response, time course, genetic perturbation, rescue or direct measurements of cell death, F-actin collapse or NADPH were available. GSE254444 represented one VHL-related case, while the GSE207493 comparator was described only as non-VHL-germline. Finally, module scores, immune-infiltration estimates and drug-response predictions are computational surrogates that require orthogonal validation. These limitations make the framework hypothesis-generating rather than a validated clinical biomarker or therapeutic target.

In conclusion, the integrated analyses identify *NPR3* and *ZNF366* as cell-type-associated candidate markers of the oxidative stress–disulfidptosis transcriptional state in ccRCC. The molecular groupings show method-dependent stability and transferable immune/stromal patterns but have not been independently validated for prognosis. The four-gene score shows modest discrimination, scale-dependent transfer and a within-ICI-arm association with clinical benefit and progression-free survival. The framework provides a testable basis for compartment-aware functional studies and for evaluating the four-gene signal alongside established clinical models, but its mechanistic and clinical value requires experimental and prospective validation.

## Data Availability

The datasets presented in this study can be found in online repositories. The names of the repository/repositories and accession number(s) can be found in the article/Supplementary Material.

## Supplementary material

SUPPLEMENTARY FIGURE S1 Kaplan-Meier survival analyses of the 21 DOS genes in the TCGA-KIRC cohort.

SUPPLEMENTARY FIGURE S2 DOS gene expression and immune infiltration patterns across gene clusters. **(A)** Expression of the 21 DOS genes among gene clusters **(A–C)**. **(B)** Immune cell infiltration differences among gene clusters **(A–C)**.

SUPPLEMENTARY FIGURE S3 Selection and evaluation of genes used for the DOS risk model. **(A)** Univariable Cox regression of candidate differential genes. **(B)** LASSO Cox cross-validation curve for model tuning. **(C)** Multivariable Cox regression identifying the final four model genes. **(D)** Time-dependent receiver operating characteristic (ROC) curves evaluating prognostic discrimination. DOS, disulfidptosis–oxidative stress; LASSO, least absolute shrinkage and selection operator.

SUPPLEMENTARY FIGURE S4 Clinical, immune, drug-sensitivity and prognostic associations of the DOS risk score. **(A)** Risk-score distributions across N, M and T stage groups. **(B)** Immune-cell infiltration differences between high- and low-risk groups. **(C)** Predicted half-maximal inhibitory concentration (IC50) values for selected agents by risk group. **(D)** Tumor Immune Dysfunction and Exclusion (TIDE) score comparison. **(E,F)** Correlations between the risk score and selected immune-cell subsets. **(G,H)** Univariable and multivariable Cox regression analyses of the risk score and clinical variables in the training set, test set and full cohort. The underlying values and source code for the original panels were unavailable, so their original test-selection and multiplicity procedures could not be reverified. Displayed P values in these panels are interpreted as exploratory.

SUPPLEMENTARY FIGURE S5
*NPR3* and *ZNF366* expression across DOS subtypes and gene clusters. **(A)** *NPR3* expression in DOS subtypes and gene clusters. **(B)** *ZNF366* expression in DOS subtypes and gene clusters.

SUPPLEMENTARY FIGURE S6 Quality control and cell-type annotation for the single-cell RNA-seq analysis. **(A)** Sample-level distributions of detected genes and mitochondrial transcript percentage. **(B)** Dot plot of canonical marker genes used to annotate major cell types.

SUPPLEMENTARY FIGURE S7 Patient-level compartment localization in the independent GSE207493 single-cell ccRCC cohort. **(A,B)** Patient-level compartment means of log-normalized *ZNF366* and *NPR3* expression. **(C,D)** Patient-level compartment means of the disulfidptosis-associated and oxidative-stress-associated transcriptional scores. Each gray line connects tumor, endothelial and immune compartment means from the same patient (n = 19); colored points show individual patients, and black horizontal segments show medians. Overall differences were assessed with two-sided Friedman tests and BH correction across four endpoints; adjusted q values and Kendall’s W are shown in each panel. Pairwise comparisons used two-sided exact paired Wilcoxon signed-rank tests with BH correction across 12 comparisons; the corresponding q values are also shown in each panel. The patient, not the cell, was the biological replicate. These are transcriptional patterns, not direct evidence of ROS abundance, disulfidptosis activity or causal gene function.

SUPPLEMENTARY FIGURE S8 Four-gene coefficient interpretation, internal robustness and external overall-survival transfer. **(A)** Original TCGA-KIRC univariable and four-gene multivariable Cox HRs with 95% CIs; blue circles and red squares denote univariable and multivariable estimates, respectively. The multivariable estimates are conditional associations given the other three predictors and are not marginal biological effects. **(B)** Lower-triangular Pearson correlation matrix in the GDC-derived complete-case cohort (n = 529; 4 of 533 primary tumors excluded for missing or invalid survival information). The observed correlations indicate moderate gene-gene collinearity. **(C)** VIFs; the dashed line at 5 is a heuristic reference, not a significance threshold, and does not negate the moderate correlations in panel B. **(D)** Median fixed-model coefficients and percentile 95% intervals from 1,000 case-bootstrap resamples. Coefficient-direction stability has no mechanistic interpretation. **(E)** Twenty repeat-level weighted mean held-out C-indices from repeated five-fold cross-validation; points show all repeats, the black horizontal segment shows their mean and the red dashed line is the bootstrap optimism-corrected C-index. The fold-level median and 2.5th–97.5th percentiles reported in the Results were calculated across all 100 held-out folds. **(F)** Cox associations of continuous four-gene scores with overall survival in E-MTAB-1980 (n = 101, 23 deaths), reported as HRs per 1-SD with 95% CIs. Blue denotes the original-scale formula, red denotes the gene-wise standardized sensitivity score, circles denote unadjusted models and squares denote pathological-T-stage-adjusted models. The standardized score assesses sensitivity to platform scaling; it is not a replacement clinical model, and its stronger association indicates scale dependence. BH-adjusted P values are shown for the four prespecified models. **(B–E)** Treat the predictors as fixed and do not reproduce upstream feature selection; panel F evaluates continuous-score rather than hard-cluster transfer. CI, confidence interval; HR, hazard ratio; VIF, variance inflation factor.
